# The prevalence and risk factors for hiatal hernia among patients undergoing endoscopy: A retrospective analysis

**DOI:** 10.15537/smj.2023.44.5.20220903

**Published:** 2023-05

**Authors:** Majid A. Alsahafi, Najla A. Alajhar, Amjad O. Almahyawi, Hadeel H. Alsulami, Wejdan A. Alghamdi, Lama A. Alharbi, Afnan S. Alsulami, Jumana T. Aljehani, Saad S. Alkhowaiter, Mahmoud H. Mosli

**Affiliations:** *From the Division of Gastroenterology, Department of Medicine (Alsahafi, Alajhar, Almahyawi, Alsulami, Alghamdi, Alharbi, Alsulami, Aljehanil, Mosli), Faculty of Medicine, King Abdulaziz University, Jeddah; and from the Division of Gastroenterology, Department of Medicine (Alkhowaiter), College of Medicine, King Saud University, Riyadh, Kingdom of Saudi Arabia.*

**Keywords:** hiatal hernia, GERD, epidemiology, gastroesophageal reflux disease

## Abstract

**Objectives:**

To determine the prevalence of hiatal hernia (HH) and its association with age, gender, and body mass index (BMI).

**Methods:**

We retrospectively included patients who underwent esophagogastroduodenoscopy (EGD) at an academic tertiary care hospital. Data were collected on the presence of HH as well as patient demographics including age, gender, and BMI. Univariate and multivariate analysis were done to determine risk factors for HH.

**Results:**

A total of 2805 patients were included in this study. The mean age was 48.6 (±18.6) years and males constituted 28.8% of the study population. The mean BMI was 29.7 (±8.6) kg/m². The prevalence of HH was 29.8% among all patients and 48.6% among those who underwent EGD for gastroesophageal reflux disease–related indications. There was no significant association between HH and female gender (OR 1.04, 95%CI: 0.88 -1.26, *p*=0.53), older age (OR 0.77, 95%CI: 0.72 - 1.06, *p*=0.19) or BMI (OR 1.07, 95%CI: 0.9 – 1.2, *p*=0.39).

**Conclusion:**

The prevalence of HH was 28.9% based on this large endoscopy-based population. We found no association between HH and gender, age, or BMI.

**G**astroesophageal reflux disease (GERD) is a common gastrointestinal disease with an estimated prevalence of 18-27% in the United Sates population.^1^ The reported prevalence of GERD in Saudi Arabia is as high as 45%.^2^ Patients with GERD may present with typical or atypical symptoms and are at risk for GERD-related complications.^3^ Gastroesophageal reflux disease impacts the patient’s quality of life and creates a significant economic burden on the healthcare system.^4^

Among the different risk factors for GERD, hiatal hernia (HH) is a well-recognized anatomical predisposing condition.^5-7^ Hiatal hernia is also a risk factor for GERD complications. It increases the risk of GERD through different mechanisms, including impaired lower esophageal sphincter pressure, transient lower esophageal sphincter relaxation, impaired esophageal acid clearance, and delayed gastric emptying.^7^ The prevalence of HH in Western populations is reportedly 14.5-22%.^8-10^ Previous studies reported potential risk factors for HH, including older age, gender, and obesity.^11-18^ The prevalence of HH reportedly increases substantially with age.^18^ Male gender has inconsistently been associated with a higher prevalence of HH as well.^11-18^

Although GERD is common in Saudi Arabia, the prevalence of HH and its risk factors have not yet been established. Thus, this study aimed to determine the prevalence of HH and assess its association with gender, age, body mass index (BMI) among a Saudi population.

## Methods

This was a retrospective study that was performed at King Abdulaziz University Hospital, an academic tertiary care center in Jeddah, Saudi Arabia. Ethical approval was obtained from the Institutional Review Board. The endoscopy database was reviewed from May 2014 to December 2018. All patients who underwent esophagogastroduodenoscopy (EGD) were included. Patients younger than 18 years were excluded. For patients who underwent more than one EGD, only the first procedure was included. The procedures were carried out by a gastroenterology consultant or a gastroenterology fellow under supervision. The presence of a sliding HH was determined endoscopically by observing the discordance between diaphragmatic impingement and the esophagogastric junction. The collected data included patient demographic variables, height, weight, and EGD indication.

### Statistical analysis

Frequencies and percentages are used to summarize the categorical variables, while mean and standard deviation are used to express continuous variables. Univariate and multivariate analyses were used to examine the association between HH and different variables, including age, gender, and BMI. Two-tailed *p*-values of <0.05 were considered statistically significant. The statistical analysis was performed using R statistical software.

## Results

A total of 2805 patients were included in the study. The mean age was 48.6 (±18.6) years, and 28.8% were older than 60 years. Male patients constituted 38.8% of the study population. The BMI data were available for 2588 patients. The mean BMI was 29.7 (± 8.6) kg/m². Table 1 shows the general characteristics of the study population.

**Table 1:** - Characteristics of the study population.

Characteristic	n	%
***Gender***		
Females	1717	61.2
Males	1088	38.8
***Age***		
≤60	1995	71.1
>60	810	28.8
***Body mass index***		
≤30	1618	62.5
>30	970	37.5

Among all patients, 837 (29.8%) were reported to have HH endoscopically. The prevalence of HH was 28.6% among men and 30.5% among women. The prevalence of HH was 30.6% among patients aged <60 years and 27.6% among those aged >60 years. For patients with a BMI <30, the prevalence of HH was 28.7%, while for obese patients (BMI >30), it was 30.6%.

For the patients who had HH, 37.2% were male, the mean age was 47.3 (± 18) years, and the mean BMI was 30.28 (± 8). For patients without HH, 39.4% were male, the mean age was 49.2 (±18) years, and the mean BMI was 29.5 (±8.6). Among 226 patients who underwent EGD for GERD-related indications (refractory GERD, screening, preoperative), the prevalence of HH was 48.6%.

Table 2 shows the univariate analysis result, while Tables 3 shows multivariate analysis result. There was no significant association between HH and gender, age >60 years, or obesity (BMI >30).

**Table 2:** - Univariate analysis of the risk factors for hiatal hernia.

Variable	n (%)	OR	95% CI	*P*-value
Female	1716 (61.2)	1.09	0.93 – 1.29	0.29
Age >60	810 (28.8)	0.86	0.72 – 1.03	0.11
BMI >30	970 (37.5)	1.10	0.92 – 1.30	0.30

BMI: body mass index, OR: odd ratio, CI: confidence interval

**Table 3:** - Multivariate analysis of the risk factors for hiatal hernia.

Variable	OR	95% CI	*P*-value
Female	1.04	0.88 – 1.26	0.53
Age > 60	0.77	0.72 – 1.06	0.19
BMI > 30	1.07	0.9 – 1.2	0.39

BMI: body mass index, OR: odd ratio, CI: confidence interval

## Discussion

Gastroesophageal reflux disease is a common medical problem worldwide for which HH is a well-recognized common risk factor. In this large study, we found the prevalence of HH was 29.8% among patients who underwent EGD for any indication and 48.7% among those who underwent EGD for GERD-related indications. The prevalence of HH in our study was slightly higher than that reported in a meta-analysis, which reported it as 20.3%.^8^ In contrast, the prevalence of HH was 49% in a UK study.^19^ The variation observed in the prevalence of these results could be attributed to differences in the included patient populations.

Although several factors are associated with the risk of developing HH, we could not establish a relationship between HH and gender, age, or BMI. Older age was previously reported as associated with HH.^18,20^ This could be caused by age-related fibromuscular degeneration and loss of elasticity of the structures surrounding the diaphragmatic hiatus.^11^ However, other studies reported no association between age and HH, similar to our findings.^21^

In our study, the prevalence of HH was similar in men (28.6%) and women (30.5%). The effect of gender on HH prevalence has not been consistent in the literature. A systematic review reported a higher prevalence of HH among men versus women (56.7% versus [vs.] 43.3%).^1^ In contrast, a higher prevalence among women was reported in a US study (66% vs. 34%).^18^ Thus, we speculate that gender may not have a direct impact on HH development; rather, other factors may have contributed to the differences.

In the present study, no association was observed between obesity, as defined by BMI, and HH, which resembles what has been reported in other studies.^21^ In contrast, a meta-analysis concluded a significant association between BMI and HH.^11^ Variations in the included populations may account for these different findings. However, abdominal obesity, as measured by waist circumference, is likely more important than BMI.^21^

### Strength and limitations

The strengths of our study include the fact that it is the largest study to date to report the prevalence of HH in the Saudi population. We acknowledge the presence of several limitations, including the inherent limitations that characterize the retrospective design and the single - center nature. Hiatal hernia details were collected from endoscopy reports, and there is likely to be variation in the observation and reporting of HH among endoscopists. We were unable to collect data on other potential risk factors for HH, such as waist circumference. Future multicenter prospective studies are needed to overcome the limitations of our study.

In conclusion, the prevalence of HH in the Saudi population was 28.9% in this large endoscopy-based population. We found no association between HH and gender, age, or BMI.
